# Dosimetric and biological comparisons of single planning and double plannings for bilateral lung cancer SBRT planning based on the Cyber-Knife system

**DOI:** 10.3389/fonc.2022.1015999

**Published:** 2022-11-21

**Authors:** Xueling Guo, Huanfan Su, Fuying Wan, XianZhi Zhao, Tingting Cao, Zhitao Dai, HuoJun Zhang

**Affiliations:** ^1^ Department of Radiation Oncology, The First Affiliated Hospital of Naval Medical University, Shanghai, China; ^2^ Department of Medical Imaging, Jiangxi Medical College, Shangrao, China; ^3^ College of Biomedical Engineering, Southern Medical University, Guangzhou, China; ^4^ Department of Radiation Oncology, Shanghai First Maternity and Infant Hospital, Tongji University School of Medicine, Shanghai, China; ^5^ Radiotherapy center, Department of Oncology, Tongji Hospital, Tongji Medical College, Huazhong University of Science & Technology, Wuhan, China; ^6^ Department of Radiation Oncology, National Cancer Center/National Clinical Research Center for Cancer/Cancer Hospital & Shenzhen Hospital, Chinese Academy of Medical Sciences and Peking Union Medical College, Shenzhen, China

**Keywords:** bilateral lung cancer, stereotactic body radiation therapy(SBRT), single planning, double plannings, Cyber-Knife system

## Abstract

**Objective:**

The aim is to investigate the influence of single planning (Plan S) and double plannings (Plan D) on bilateral lung cancer stereotactic body radiation therapy planning from the perspective of dosimetry and biology respectively. Methods Cases with bilateral lung cancer patients who had undergone SBRT with the Cyber-Knife were enrolled, and a single planning and double plannings were designed in the Multiplan@4.2 treatment planning system equipped with the Cyber-Knife system. The single plan was to optimize the two target volumes in a separate plan, while the dual plan is to optimize two target volumes respectively in two separate plans, then perform dose superposition. Then based on the dosimetric results, the biological parameters were calculated. Thus the quality of SBRT plans for those bilateral lung cancer designed by the two methods were compared and evaluated according to the dosimetric and biological results.

**Results:**

The dose distribution of both planning target volumes and surrounding organs at risk in Plan S and Plan D could meet the clinical prescription requirements. The target conformity index and the new conformity index of PTV were closer to 1 in the Double plannings, and the dose gradient GI in the Plan D was smaller than Plan S. For organs at risks, the doses received by the Plan D were relatively small. In terms of biological models, for the equivalent uniform dose of normal lung tissue, heart and esophagus, the Plan D was 6.51% (*P*=0.045), 19.8% (*P*=0.022), 27.08% (*P*>0.05) lower than Plan S respectively. The results showed that the equivalent uniform dose of normal tissue in the Plan D was lower relative to Plan S.

**Conclusions:**

Dosimetric and biological results show that both the use of Plan D have an advantage of protecting normal tissues, and it was suggested that to design double plannings for bilateral lung cancer stereotactic body radiation therapy planning based on Cyber-Knife in the clinical practice.

## Introduction

Cancer is a major public health problem worldwide, of which lung carcinoma is one of the most common types of malignant tumor, and is the leading cause of cancer death in China and USA in both males and females (1, 2). Multiple primary lung cancers (MPLC) were first proposed and described in 1920s (3). These represent an interesting subgroup of cancer cases which may occur after curative resection of bronchogenic carcinoma (4). Incidences were reported from 0.5% to 10% in lung cancers (4), and they may appear as unilateral or bilateral, synchronous or metachronous.

The feasibility of surgery was reported earlier (3, 4), but with the update of radiotherapy technology, more and more reports have been reported where radiotherapy was used in the treatment of MPLC, especially for inoperable patients. Federico et al. (5) believed that palliative radiotherapy could provide an acceptable symptom-free quality of life for synchronous bilateral lung cancer patients who may not be suitable for surgery in 2001, although with poor median overall survival. Loo et al. (6) suggested that intensity-modulated radiotherapy (IMRT) could be considered as an effective treatment for synchronous bilateral cancers, unfortunately from only one case with a Varian 2100EX linear accelerator system. Sinha et al. (7) believed that stereotactic body radiation therapy (SBRT) based on linac accelerator may be a possibly safe and potentially effective treatment option for individuals with bilateral lung cancers who were medically inoperable, by reviewing the outcomes of 10 patients.

In recent years, stereotactic body radiation therapy (SBRT) has been applied as a clinical treatment option in the treatment against some lung cancers (8–12). SBRT is characterized by the delivery of high doses of ionizing radiation in few fractions, which results in a higher biological effective dose (BED), and better tumor control probability (TCP) and lower normal tissue complication probability (NTCP). The radiotherapy equipment that can perform SBRT, which can be performed either with a traditional linear accelerator or a robotic arm (Cyber-Knife^®^), would generally more accurately track the movements of tumors and take corresponding compensation methods, so SBRT should be more accurate than traditional radiotherapy (13–16).

With the invention of the Cyber-Knife (Accuray incorporated, sunnyvale, CA, USA), because of its powerful function such as the precise image-guided radiotherapy through a dual kV X-ray imaging system and the capacity for real-time monitoring tumors, which can be achieved with a Synchrony^Ⓡ^ system (17), it has been increasingly employed for SBRT of lung cancers (18, 19).

In clinical practice, the design of an SBRT plan for synchronous bilateral lung cancer can be implemented in two ways based on a Cyber-Knife system. One of them is to superimpose two targets into an overall target structure, and then complete the dosage optimization of two separate targets in only one plan, this optimization method is called single planning (Plan S); the other is to optimize the dosage of the two targets separately, which means that two plans need to be completed independently, and then superimpose the dosage of the two plans, this optimization method is called double plannings (Plan D). The authors have not found any comparative study on the dosimetry of Plan S and Plan D for synchronous bilateral lung cancer. Therefore, in this study, we aimed to compare the dosimetric and biological difference of single planning and double plannings for synchronous bilateral lung cancer SBRT planning based on Cyber-Knife, and hence provide reference for clinical practice.

## Materials and methods

### Simulation

Ten patients with bilateral lung cancer treated by Cyber-Knife (Accuray incorporated, sunnyvale, CA, USA) at our institution were retrospectively evaluated for this study. These patients all had two lung cancer lesions, one on each side of the left and right lungs. All belongs to the peripheral lung cancer. The computed tomography (CT) images of 1.5 mm thickness were acquired on a GE Discovery Big bore CT simulator in the head first-supine position; after acquiring the CT images, the patient’s magnetic resonance (MR) images were acquired with a 1.2mm slice thickness and T2/T1 scan sequence was selected. Then the CT images and MR images were transmitted to the Multiplan planning system (version 4.2) and image fusion was performed at the same time.

### Target volume delineation

Experienced oncologist and radiologist outlined the gross tumor volume GTV1 and GTV2 of synchronous bilateral lung cancer on the fusion images. Taking into account the uncertain factors such as breathing movement, organ movement and positioning errors, we expanded 5mm margin of GTV1 and GTV2 in all directions to set the planning target volume PTV1 and PTV2. The median volume of PTV1 and PTV2 were 16.93cm^3^ (1.67~62.31cm^3^), 7.375cm^3^ (4.49~26.21cm^3^), respectively. In order to evaluate the dose distribution of the entire target area, PTV1 and PTV2 need to be combined to form PTV12, the median volume of which is 24.455 cm^3^ (8.06~83.07cm^3^).

All the organs at risk (OARs) were contoured to ensure the incidental radiation delivered to the structures was limited to meet the clinical requirements, including lung, heart, spinal cord, trachea, bronchus, esophagus, etc.

### Treatment planning procedures

After the delineation of the targets areas and the OARs, the Multiplan@4.2treatment planning system (TPS) was used to design the two SBRT plans based on the same set of CT images. One of them is to superimpose two targets into an overall target structure, and then complete the dosage optimization of two separate targets in only one plan, this optimization method is called single planning (Plan S); the other is to optimize the dosage of the two targets separately, which means that two plans need to be completed, and then superimpose the dosage of the two plans, we named it as double plannings (Plan D). We use the spine-tracking method for these patients, and chose one path for delivering the beams. As to the collimator, we choose the Conformality option, but not the Homogeneity. The prescribed dose to both PTV1 and PTV2 were 50Gy delivered in 5 fractions. A 6 MV photo beam was used, the dose rate was 800MU/min, and the sequential optimization method based on the ray tracing algorithm was used (20). There are two options on the Multiplan TPS for dose calculation, ray tracing algorithm and the Monte Carlo algorithm. The Monte Carlo (MC) algorithm model the actual physical processes including secondary electron distributions (21), that is recognized as the most accurate methods of dose calculations available, but it is very time-consuming, about 25 times (20) than the ray tracing algorithm. Though the MC algorithm would cause some dose changes in different ROIs, it would not affect the conclusion of this study. The dosimetric requirements and constraints for PTV and OARs are shown in **Table 1**.

**Table 1:** Dosimetric requirements and constraints for PTV and OARs.

Structure	Parameter	Objective
PTV	V_100_ (%)	≥95%
	PIDL (%)	~70%
Combined lungs	V_5Gy_ (%)	<60%
	V_20Gy_ (%)	<25%
	V_<12.5Gy_ (cc)	>1500cc
	V_<13.5Gy_ (cc)	>1000cc
Heart	D_max_ (Gy)	<38Gy
	D_mean_ (Gy)	<12Gy
	V_32Gy_ (cc)	<15cc
Spinal cord	D_max_ (Gy)	<27Gy
	D0.25cc (Gy)	<22Gy
	D1.2cc (Gy)	<13.5Gy
Trachea	D_max_ (Gy)	<40Gy
	V_16.5Gy_ (cc)	<4cc
Bronchus	D_max_ (Gy)	<40Gy
	V_16.5Gy_ (cc)	<4cc
Esophagus	Dmax (Gy)	<35Gy
	V_19.5Gy_ (cc)	<5cc

### Dosimetric and biological parameters for evaluation

The evaluation parameters mainly include the coverage of PTV,the maximum dose(D_max_), the mean dose(D_mean_), the conformity index(CI) (22), the new conformity index(nCI) (23), the homogeneity index(HI) (24) and the gradient index(GI) (25), etc., where the coverage was defined as the percentage of the volume included in the prescribed dose line in the targets to the total volume of the targets. For the organs at risk, the combined lungs mainly involve the average dose (D_mean_),V_5_,V_20_,V_<12.5Gy_,V_<13.5Gy_, etc., and V_x_ represents the volume of the corresponding organ receiving x Gy; the heart mainly involves D_mean_, D_0.25cc_, D_1.2cc_, D_x cc_ represents the dose received by the volume of x cm^3^ in the corresponding organ; trachea and bronchus mainly involves D_max_,D_mean_, etc., when combining the trachea and bronchus into one structure, it is also necessary to evaluate D_4cc_ in addition to D_max_ and D_mean_; esophagus mainly involves D_max_, D_mean_, D_5cc_, etc.

The concept of equivalent uniform dose (EUD) assumes that different dose distributions are equivalent if they are able to elicit the same radiobiological effect (26, 27). For the biological parameters, the calculation of the equivalent uniform dose (EUD) for each structure should be based on the revised biological model (28), the parameters in the EUD model are shown in **Table 2**. Here *a* is a parameter which reflects the dose response property of distinct organs. The *α/β* is a parameter from the issue-specific linear quadratic (LQ) model of the certain organ, which could determine the fractionation sensitivity.

**Table 2:** Parameters of each structure in EUD model.

Structure	*a*	α/β
PTV	10	10
Lung	1	4
Heart	3	3.7
Spinal cord	20	3
Esophagus	16.67	4.9

### Statistical analysis

All the analyses were performed using the SPSS 24.0 software (SPSS Inc.,Chicago,IL,USA). Paired *t*-test was performed based on the data results between the Plan S and the Plan D, *p*<0.05 was considered to indicate a statistically significant difference.

## Results

**Figure 1:**
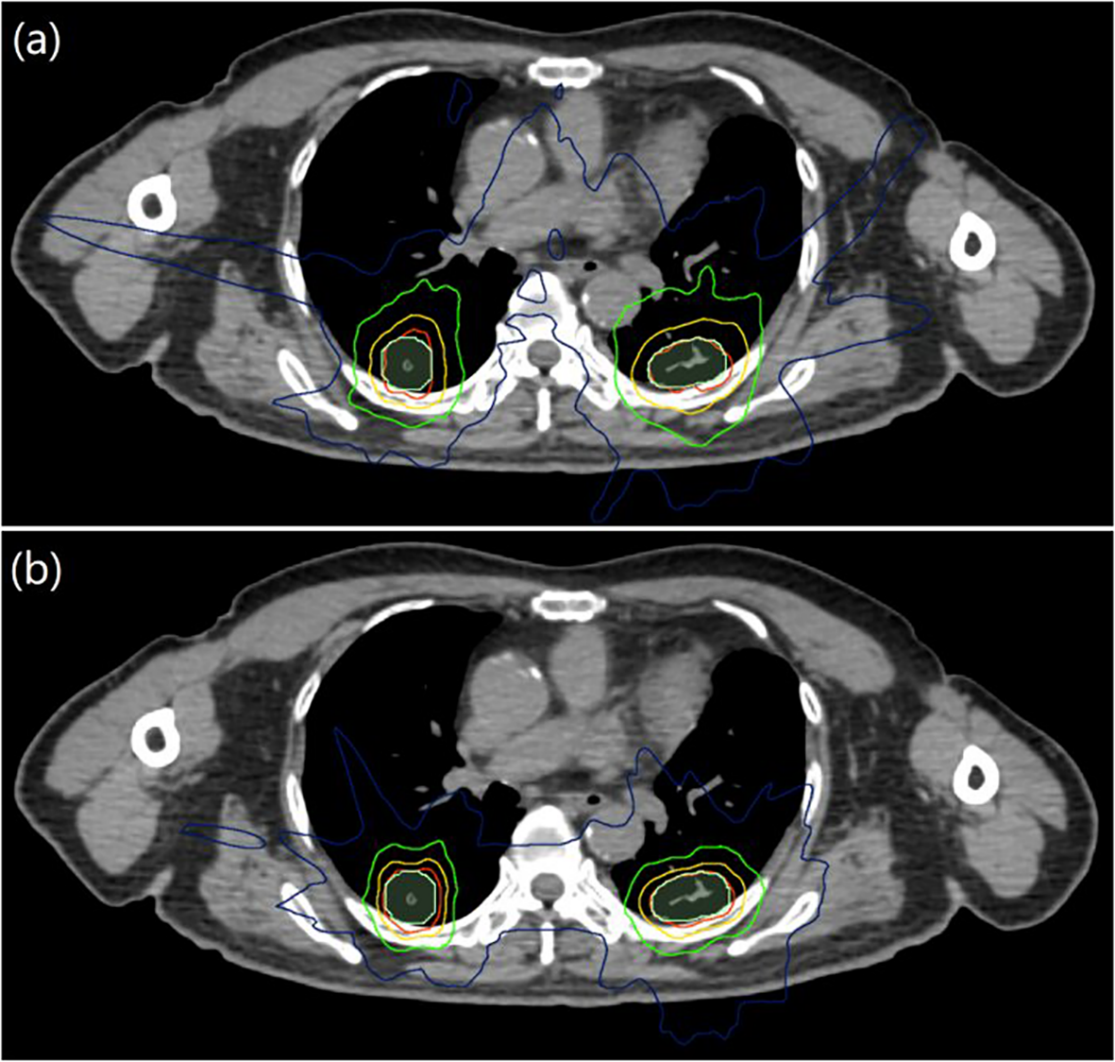
Transverse section isodose distributions for Plan S and Plan D of synchronous bilateral lung cancer, **(A)** was Plan S, **(B)** was Plan D; PTV was represented by light green area, and the red, yellow, green and blue lines were isodose lines of 50, 40, 25 and 10 Gy, respectively.

The results showed that the dosimetry of the Planning target volumes (PTVs) and the OARs for synchronous bilateral lung cancer SBRT plans designed by both Plan S and Plan D could meet the clinical prescription requirements. The transverse section isodose line distribution of the two plans is shown in **Figures 1A, B**, it can be seen from the figure that different plans can well surround the target areas. However, for the isodose line of 40Gy, 25Gy, 10Gy, the Plan D tends to involve less tissue volume relative to Plan S, especially for the low-dose isodose lines (25Gy and 10Gy). Therefore, the Plan D would irradiate less normal tissues. We compared the two planning methods from the dosimetric and biological parameters, and the results were as follows.

### Dosimetric comparisons

**Figure 2:**
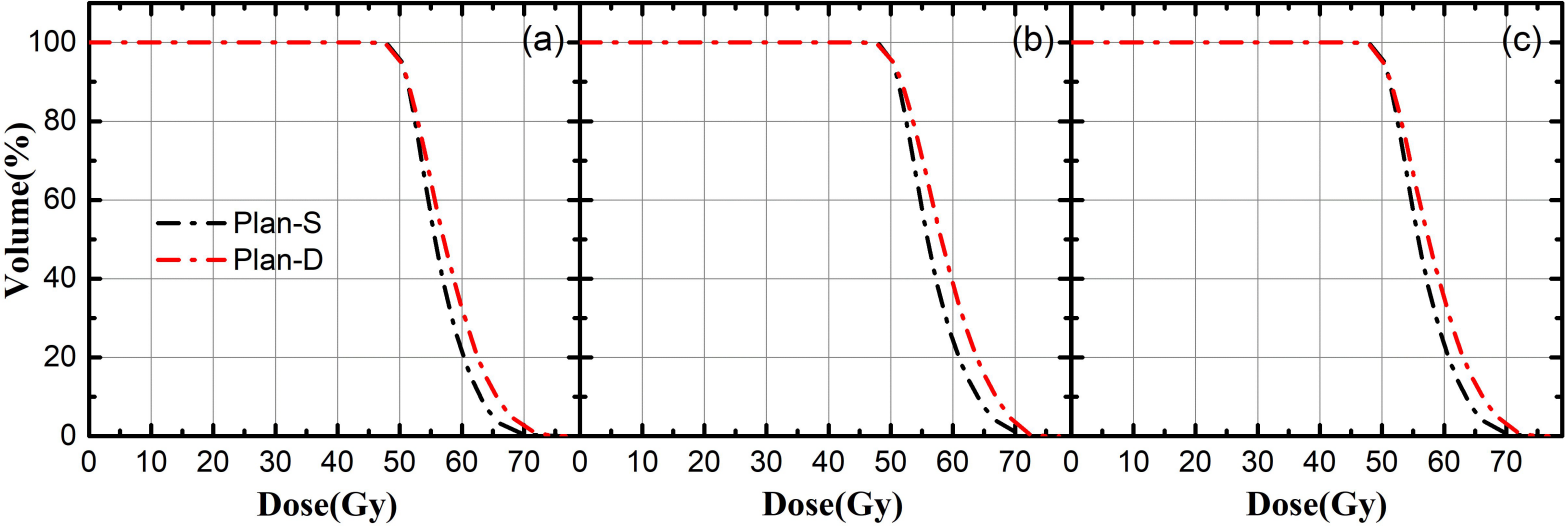
The average dose volume histogram of PTV; **(A)** represents PTV1, **(B)** represents PTV2, **(C)** represents PTV12; black line indicate Plan-S, red line indicate Plan-D.

**Figure 2** shows the average dose volume histogram of planning target volumes (PTVs) for the two different planning methods for synchronous bilateral lung cancer, from (a) to (c) stands forPTV1,PTV2 and PTV12, respectively. All the coverage of PTV1, PTV2, PTV12 can meet the requirements of clinical prescription for both Plan S and Plan D. However, the high dose in PTVs of Plan D are relatively larger, and the PTVs falls faster in high dose area for Plan S, this is mainly due to the overlapping of the background doses in Plan D.

The dosimetric results of different PTVs based on Plan S and Plan D for synchronous bilateral lung cancer were shown in the **Table 3**. Compared with the Plan D, the coverage of PTV1, PTV2, PTV12 obtained from Plan S are relatively higher, which are respectively 95.58 ± 0.90, 96.14 ± 1.55, 95.83 ± 0.65(%), the differences are not statistically significant (*P*>0.05).

**Table 3:** Comparisons of dosimetric parameters for different PTVs in synchronous bilateral lungs.

Targets	Parameter	Plan S	Plan D	*P*
		Mean ± SD	Mean ± SD	
**PTV1**	Coverage (%)	96.32 ± 1.56	95.58 ± 0.90	0.155
	D_max_ (Gy)	70.21 ± 4.12	72.89 ± 2.06	0.050
	D_min_ (Gy)	46.05 ± 1.18	45.20 ± 1.42	0.023
	D_mean_ (Gy)	56.42 ± 1.50	57.83 ± 1.33	0.032
	CI	1.20 ± 0.13	1.14 ± 0.08	0.100
	nCI	1.25 ± 0.14	1.19 ± 0.08	0.130
	HI	1.40 ± 0.08	1.46 ± 0.04	0.050
	GI	5.76 ± 1.32	5.19 ± 0.81	0.070
**PTV2**	Coverage (%)	96.18 ± 1.53	96.14 ± 1.55	0.962
	D_max_ (Gy)	71.74 ± 3.78	73.38 ± 1.61	0.248
	D_min_ (Gy)	45.86 ± 1.04	46.76 ± 3.83	0.434
	D_mean_ (Gy)	56.82 ± 1.48	58.75 ± 1.47	0.006
	CI	1.19 ± 0.15	1.23 ± 0.14	0.647
	nCI	1.24 ± 0.14	1.27 ± 0.13	0.596
	HI	1.43 ± 0.08	1.47 ± 0.03	0.248
	GI	6.28 ± 1.25	5.29 ± 0.70	0.036
**PTV12**	Coverage (%)	96.42 ± 0.83	95.83 ± 0.65	0.056
	D_max_ (Gy)	73.22 ± 2.22	74.42 ± 1.58	0.184
	D_min_ (Gy)	45.22 ± 0.89	44.74 ± 1.26	0.162
	D_mean_ (Gy)	56.68 ± 1.26	58.17 ± 0.98	0.013
	CI	1.19 ± 0.13	1.16 ± 0.06	0.422
	nCI	1.24 ± 0.13	1.21 ± 0.06	0.537
	HI	1.46 ± 0.04	1.49 ± 0.03	0.184
	GI	5.85 ± 1.03	5.18 ± 0.58	0.031

For the target index, we compared the D_max,_ D_mean_, D_min_, and CI, nCI, HI, and GI as mentioned above in the part of the Dosimetric and biological parameters for evaluation. The results show that the D_max_ and D_mean_ of PTVs from the Plan D are higher than Plan S, while the D_min_ is lower than Plan S. The results show that the CI and nCI of PTVs in the Plan D are more closer to 1, which indicate that the prescription dose line can better conform to the target areas, but the difference between the two plans is not statistically significant (*P*>0.05). For the HI, the dose uniformity of the targets in two plans is similar, and the difference of HI in the two plans is not statistically significant (*P*>0.05). For the GI, the GI value in Plan D is smaller, indicating that the dose outside the target areas falls faster, which means that the Plan D can better protect the OARs around the targets, and the differences between Plan S and Plan D are statistically significant (*P*<0.05) for PTV2 and PTV12.

**Figure 3:**
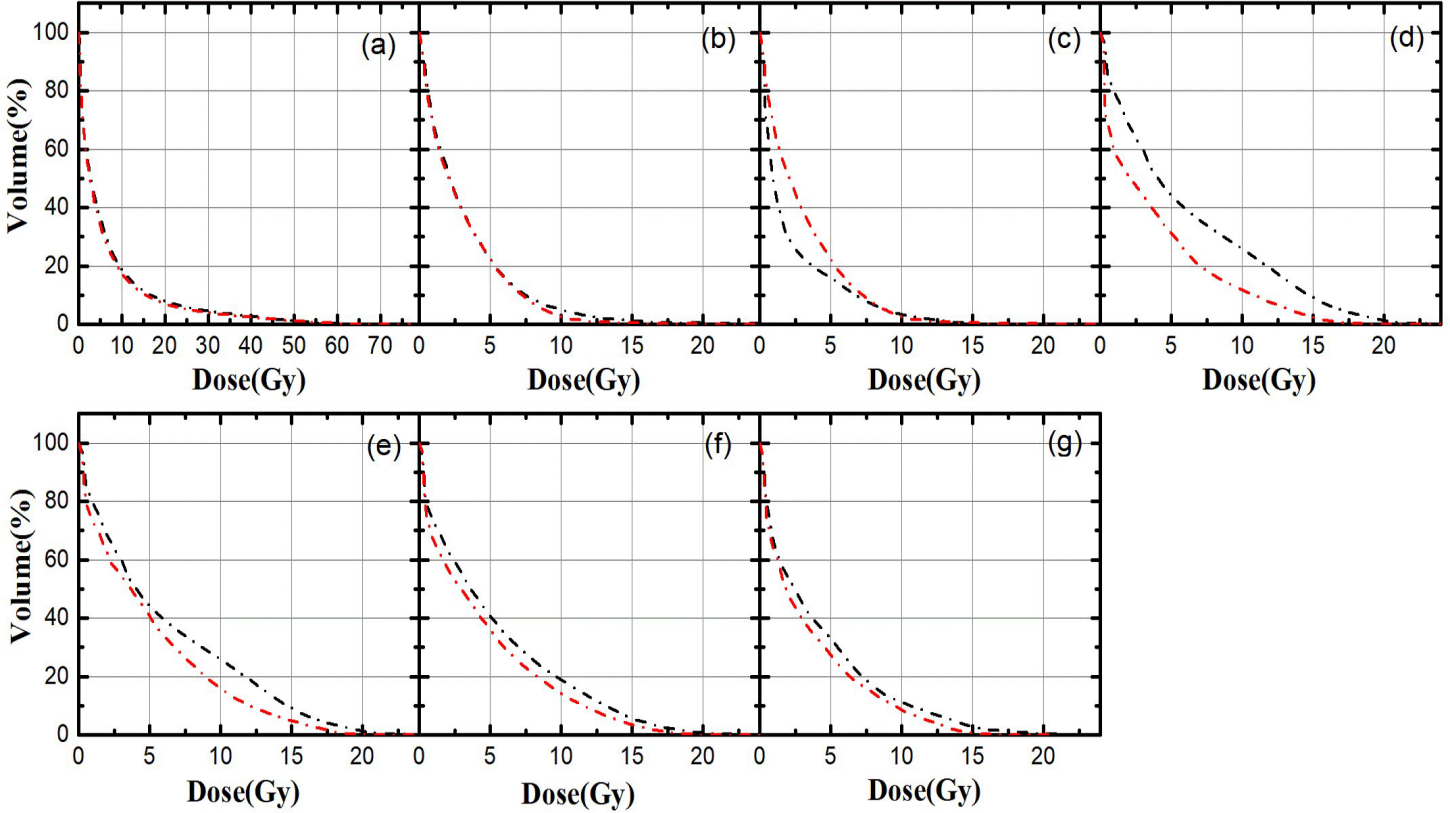
The average dose volume histograms of OARs in Plan S and Plan D; **(A)** combined lungs, **(B)** heart, **(C)** spinal cord, **(D)** trachea, **(E)** bronchus,**(F)** combined the trachea and bronchus, **(G)** esophagus; black line indicate Plan S, red line indicate Plan D.

**Figure 3** shows that the average dose volume histograms (DVHs) of the OARs in the two SBRT plans, (a)~(g) are combined lung, heart, spinal cord, trachea, bronchus, combined the trachea and bronchus, esophagus, etc. For the combined lungs and heart, the differences between Plan S and Plan D are not statistically significant(*P*>0.05). As for the trachea, bronchus and esophagus, the dosage in Plan D is relatively smaller.

**Table 4** shows the dosimetric comparisons of the OARs for Plan S and Plan D. For the combined lungs, the difference of D_mean_ between Plan S and Plan D is tiny; but the for V_5_ and V_20_, the radiation exposure volume of corresponding dose in Plan D is less, which are 37.06 ± 20.35, 7.16 ± 4.84(%), respectively, while in Plan S, they are 38.78 ± 17.70, 8.01 ± 5.07(%), respectively. For the protected lung, the V<12.5Gy (cc) and V<13.5Gy (cc) in the Plan D are both larger than in the Plan S. Therefore, we can think that the Plan D can better protect the healthy lungs. In details, for the normal lung tissue, the V_5_ and V_20_ of the Plan D was 4.43% (P>0.05), 10.61% (P=0.017) lower than the Plan S respectively. For the heart, spinal cord, trachea and bronchus, the Plan D were smaller than single planning. For the heart, the D_mean_ of Plan D is relatively lower. For the spinal cord, the difference of D_mean_ between Plan S and Plan D is not statistically significant (*P*>0.05), but the values of D_0.25cc_ and D_1.2cc_ in Plan D are smaller relative to Plan S (*P*>0.05). For the trachea and bronchus, the D_max_, D_mean_ and D_4cc_ in Plan D are smaller than those in Plan S. For the esophagus, the D_mean_, D_max_ and D_5cc_ in Plan D are 3.33 ± 1.91, 12.7 ± 4.94, 7.96 ± 3.38(Gy), respectively, while in Plan S they are 4.09 ± 1.91, 15.95± 6.26, 9.75 ± 4.04(Gy), respectively, the results further illustrate that Plan D has better protection for normal tissues and can ensure adequate dose distribution in the target area.

**Table 4:** Dosimetric comparisons of the OARs for Plan S and Plan D.

Structure	Parameter	Plan S	Plan D	*P*
		Mean ± SD	Mean ± SD	
**Combined lungs**	D_mean_ (Gy)	6.61 ± 3.08	6.21 ± 2.96	0.082
	V_5_ (%)	38.78 ± 17.70	37.06 ± 20.35	0.951
	V_20_ (%)	8.01 ± 5.07	7.16 ± 4.84	0.017
	V_<12.5Gy_ (cc)	2574.08 ± 833.09	2598.05 ± 827.63	0.096
	V_<13.5Gy_ (cc)	2608.84 ± 829.77	2649.26 ± 847.18	0.05
**Heart**	D_mean_ (Gy)	3.26 ± 1.63	3.04 ± 1.60	0.333
	D_15cc_ (Gy)	11.11 ± 5.09	9.49 ± 3.69	0.049
**Spinal cord**	D_mean_ (Gy)	2.25 ± 1.37	2.36 ± 1.42	0.406
	D_0.25cc_ (Gy)	9.76 ± 4.77	9.235 ± 4.09	0.506
	D_1.2cc_ (Gy)	8.34 ± 4.30	8.15 ± 3.73	0.776
**Trachea**	D_mean_ (Gy)	4.21 ± 2.17	3.86 ± 3.15	0.328
	D_max_ (Gy)	12.69 ± 8.79	11.51 ± 7.43	0.354
**Bronchus**	D_mean_ (Gy)	6.11 ± 4.60	4.96 ± 3.81	0.012
	D_max_ (Gy)	19.82 ± 9.31	16.07 ± 9.14	0.003
**Combined the trachea** **and bronchus**	D_mean_ (Gy)	5.23 ± 3.45	4.44 ± 3.03	0.068
	D_max_ (Gy)	20.17 ± 9.48	16.51 ± 8.54	0.011
	D_4cc_ (Gy)	11.79 ± 6.34	10.47 ± 5.54	0.066
**Esophagus**	D_mean_ (Gy)	4.09 ± 1.91	3.33 ± 1.91	0.068
	D_max_ (Gy)	15.95 ± 6.26	12.7 ± 4.94	0.013
	D_5cc_ (Gy)	9.75 ± 4.04	7.96 ± 3.38	0.064

### Biological comparisons

**Table 5** shows the calculation results of equivalent uniform dose (EUD) for the PTVs and OARs. For PTV1, PTV2, PTV12, they are respectively 94.18 ± 1.67,94.58 ± 1.91, 94.41 ± 1.51 (Gy) in Plan S,and 95.18 ± 0.93, 96.97 ± 1.83, 95.55 ± 0.67 (Gy) in Plan D, indicating that the EUD of the targets in Plan D is higher, but the difference is not statistically significant (*P*>0.05). For the combined lungs, the EUD values of Plan S and Plan D are respectively 9.21 ± 4.12, 8.61 ± 3.97 (Gy), which shows that the Plan D has a lower EUD for the combined lungs, there is statistically significant difference(*P*=0.049). For the heart, the EUD values of Plan S and Plan D are respectively 6.21 ± 3.15,4.98 ± 2.38 (Gy), which also show that the EUD value in Plan D is lower, and the difference is statistically significant(*P*=0.022). For the spinal cord, the EUD of Plan S and Plan D are 8.80 ± 4.48, 8.02 ± 3.64 (Gy) respectively, and the difference is not statistically significant (*P*>0.05). For the esophagus, the EUD of Plan S and Plan D are 13.33 ± 5.58, 9.72 ± 4.30 (Gy) respectively, and the difference is not statistically significant(*P*>0.05). The above results indicate that the equivalent uniform dose (EUD) of the organs at risk (OARs) in Plan D is relatively lower.

**Table 5:** Comparisons of equivalent uniform dose (EUD) for the PTVs and OARs.

Structure	EUD(Mean ± SD)(Gy)		*P*
	Plan S	Plan D	
**PTV1**	94.18 ± 1.67	95.18 ± 0.93	0.229
**PTV2**	94.58 ± 1.91	96.97 ± 1.83	0.105
**PTV12**	94.41 ± 1.51	95.55 ± 0.67	0.140
**Combined lungs**	9.21 ± 4.12	8.61 ± 3.97	0.049
**Heart**	6.21 ± 3.15	4.98 ± 2.38	0.022
**Spinal cord**	8.80 ± 4.48	8.02 ± 3.64	0.471
**Esophagus**	13.33 ± 5.58	9.72 ± 4.30	0.099

## Discussion

The robotic arms of the Cyber-Knife system can focus radiation rays from different directions to the target areas, and it has accurate image-guided technology, and has advanced tumor tracking system Synchrony Respiratory (Accuray Inc.,Sunnyvale, CA), these functions all can be used to compensate for the effect of tumor movement due to breathing (29); Cyber-Knife can also use the optical imaging equipment to track the movement of external markers to predict the position of the tumor, guiding the radiation to the tumor (30); the equipped KV X-ray imaging system can realize real-time correction of positioning and treatment errors during the treatment process, and realize sub-millimeter-level high-precision treatment (31),the superior performance of Cyber-Knife enables it to meet the basic requirements of implementing SBRT technology. This study is based on the fifth-generation Cyber-Knife system that uses single planning (Plan S) and double plannings (Plan D) to design SBRT plans for synchronous bilateral lung cancer. The results showed that the dosimetry of both PTVs and OARs for synchronous bilateral lung cancer SBRT plans designed by Plan S and Plan D could both achieve the clinical prescription requirements. The results show that the dose distribution of PTV1 and PTV2 for Plan S and Plan D are similar, but the hot spots of PTV1 and PTV2 in Plan D are relatively higher. Regarding the CI, HI, GI values of the targets, the results show that the CI, GI values of the targets in Plan D are relatively smaller, while the HI value is relatively larger, which indicates that the Plan D has better dose conformity and the dose outside the targets falls faster, but the dose uniformity in the targets is relatively poorer. For the organs at risk(OARs), the results show that the combined lungs, heart, spinal cord, trachea, bronchus and esophagus have lower volume doses, indicating that Plan D can better protect normal tissues. The dosage of the OARs in Plan D is lower, this is mainly because the Plan D method is to optimize the dose separately for each target, the total number of radiation fields is larger and spatial freedom is greater, which can make the dose distribution better. As a result, the dose can be focused on the target areas, so Plan D can better protect the normal tissues compared to Plan S. According to the calculation results of the EUD, it can be found that the EUD values of the PTVs in Plan D is larger, and the EUD values of combined lugs, heart, spinal cord and esophagus are smaller, so the results indicate that the targets can receive a larger radiation dose and can better protect the normal tissues in Plan D. In this study, we have not found the suitable biological model parameter values for the trachea and bronchus, so their EUD values were not calculated in the article. Only ten patients with synchronous bilateral lung cancer were selected for prospective exploration in this study, so the number of sample is relatively small. And the biological model and model parameters of this study are based on the earlier studies, so the accuracy of the parameters of different tumors and normal tissues needs further research.

Both the dosimetric and biological results in this paper indicate that the Plan D approach in SBRT for synchronous bilateral lung cancer can better protect normal tissues and is relatively safer in clinical radiotherapy applications. Although Plan D shows safer advantages in both dosimetric and biological parameters, Plan D means that two plans need to be designed, which may result in more monitor units (MU) in the actual dose transmission, and more treatment time would be required in Plan D relative to Plan S. The Plan D may take 15 more minutes, and deliver 44 more beams in average than the Plan S. And Plan D approach may increase the complexity of quality assurance (QA) and quality control (QC). Since this study is based on the Cyber-Knife system to design SBRT plans for synchronous bilateral lung cancer, the actual treatment is to complete the irradiation in two separate plans for Plan D, so the Cyber-Knife can track and locate one tumor at a time, the accuracy of tumor location would be higher; for Plan S, the special algorithm of Cyber-Knife system makes it impossible to track the movement of two separate tumors at the same time, which may affect the accuracy of tumor positioning.

One of the advantages of Cyber-Knife is that it can perform image-guided radiotherapy effectively, so assuming that the pros and cons of the two planning methods are not considered from the perspective of dosimetry and biology, but only from the aspect of powerful image-guided function for Cyber-Knife, it is better to design SBRT plan with Plan D method for synchronous bilateral lung cancer. However, synchronous bilateral lung cancer in clinical practice is rare, when Cyber-Knife is used for SBRT treatment, it would mean longer treatment time and almost double radiotherapy costs for Plan D. Therefore, in order to improve the efficiency of treatment and reduce the cost of radiotherapy for patients, some Cyber-Knife radiotherapy centers may actually adopt the Plan S approach to design SBRT plans for synchronous bilateral lung cancer. Unfortunately, from the perspective of dosimetry and biology, this study has concluded that the Plan D method has more advantages in theoretical value than the Plan S method, in order to further provide dosimetric and biological theories and supports for adopting Plan D approach to design SBRT plans for synchronous bilateral lung cancer, this is also the purpose of the author to carry out the research.

## Conclusion

In summary, the dosimetric and biological comparison between Plan S and Plan D based on Cyber-Knife for synchronous bilateral lung cancer showed that double plannings (Plan D) can better protect normal tissues, and it can ensure that the target volumes have enough dose distribution, therefore, it was suggested to design double plannings (Plan D) for synchronous bilateral lung cancer SBRT planning based on Cyber-Knife in clinical practice.

## Data availability statement

The raw data supporting the conclusions of this article will be made available by the authors, without undue reservation.

## Author contributions

XG, HS, FW, have contributed equally to this work and share first authorship. HZ and ZD have contributed equally to this work and share the corresponding authorship. All authors contributed to the article and approved the submitted version.

## Funding

This study is sponsored by 234 discipline peak-climbing plan of First Affiliated Hospital of Navy Medicial University (2019YPT004). Shenzhen Postdoctoral Research Funds (25005), Basic and Applied Basic Research Foundation of Guangdong Province (2020A1515110335), Sanming Project of Medicine in Shenzhen (SZSM201612063) and Shenzhen Key Medical Discipline Construction Fund (SZXK013).

## Conflict of interest

The authors declare that the research was conducted in the absence of any commercial or financial relationships that could be construed as a potential conflict of interest.

## Publisher’s note

All claims expressed in this article are solely those of the authors and do not necessarily represent those of their affiliated organizations, or those of the publisher, the editors and the reviewers. Any product that may be evaluated in this article, or claim that may be made by its manufacturer, is not guaranteed or endorsed by the publisher.
